# Ocular toxicity by latex of *Calotropis procera* (Sodom apple)

**DOI:** 10.4103/0301-4738.49402

**Published:** 2009

**Authors:** Samar K Basak, Arup Bhaumik, Ayan Mohanta, Prashant Singhal

**Affiliations:** Disha Eye Hospitals and Research Centre, Barrackpore, West Bengal, Kolkata - 700 120, India

**Keywords:** *Calotropis procera*, latex, ocular toxicity

## Abstract

We report the spectrum of ocular toxicity following accidental inoculation of latex of *Calotropis procera* (Sodom apple) in 29 eyes between January 2003 and December 2006. All patients presented with sudden painless dimness of vision with photophobia. Twenty-five (86%) patients had initial visual acuity of less than 20/60. All eyes had conjunctival congestion and mild to severe corneal edema with Descemet's folds. Three (10%) eyes had an epithelial defect, nine (31%) had iridocyclitis, and seven (24%) had associated secondary glaucoma. After treatment with topical corticosteroids, antiglaucoma agents, cycloplegics, hypertonic saline and tears supplements, 27 (93%) eyes recovered completely within 3–14 days. After three months, 17 (74%) out of 23 eyes showed a significant low endothelial cell count compared to the normal fellow eye (*P* < 0.001).

The latex of *Calotropis procera* causes significant ocular morbidity which may be preventable by simple health education. The long-term effect on corneal endothelium has to be studied further.

*Calotropis procera* is a member of the milkweed or *Asclepiadeae* family and it is the scientific name of Sodom apple or Madar shrub. In India, it is found mainly in Assam, West Bengal, Rajasthan, Punjab, particularly in the wastelands.[1] They are medium-branched and perennial shrubs or small trees that grow up to a height of 4–5 meters with milky latex throughout. It has white or pink flowers which bloom between February and June [Fig. 1a].[2] In Bengali, it is known as *Akanda* and in Hindi as *Ak.* Its flower or garland and the leaves are used to worship *Lord Shiva* during the festive season in the months of February and April every year in West Bengal [Fig. 1b]. Thus, it is not so uncommon to get ocular injuries caused by accidental contact or inoculation of the latex of *Calotropis procera* during plucking of the flower or leaf stalk during this season.

**Figure 1a:**
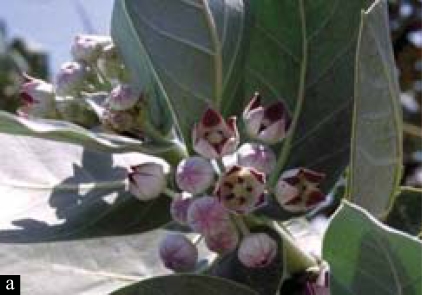
Flower and leaves of *Calotropis procera*

**Figure 1b:**
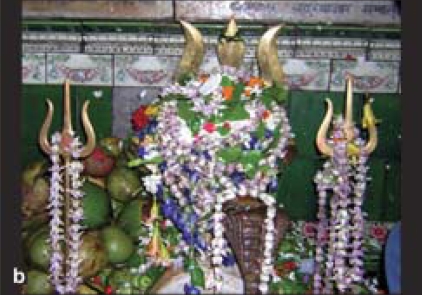
Garland of *Calotropis procera* flowers to worship Lord Shiva

The first report on ocular injury by *Calotropis* was by Muthayya in 1949 from India.[3,4] Since then, there are only three isolated case reports in literature - one from Israel, one from Thailand and the last one from Saudi Arabia.[5–7] Otherwise, the literature is silent about this kind of accidental toxic ocular injury with a large number of patients. In this communication, we report the spectrum of ocular toxicity after accidental inoculation of the latex of *Calotropis procera* in a series of patients, with their management, and further report its long-term effect on corneal endothelium.

## Case Report

A retrospective analysis of 29 patients who presented with accidental ocular contact or injury with the latex of *Calotropis procera,* in our cornea department between January 2003 and December 2006, was performed. Apart from the demographic profile, a more detailed history was obtained from each patient to determine the mode of injury. Ocular examinations like best corrected visual acuity (BCVA) on presentation, slit-lamp findings, conjunctival injection, extent of corneal involvement including fluorescein staining and intraocular pressure by non-contact tonometer (NCT) [*CT-80*,Topcon Inc, Tokyo, Japan] were carried out for each patient.

Though most of the patients had given the history of washing their face and eyes with water, further thorough wash was given to all of them by Ringer's lactate solution for those who had presented within 24 h. They were treated with tapering doses of topical antibiotic, steroids, cycloplegics, 5% hypertonic saline and oral vitamin C. Additionally, the patients with secondary glaucoma were treated with topical antiglaucoma medication and the eyes with epithelial defect with tear substitutes. All patients were followed up at a regular interval depending upon the severity of injury, and studied for all the parameters. After completion of treatment, specular microscopy (SP-*2000P*, Topcon Inc, Tokyo, Japan) was performed for the affected and fellow normal eye. The patients were asked to follow up after three to six months for routine check up and a repeat specular microscopy. The patients were taught about the toxicity of latex of *Calotropis* plant and the importance of washing hands after handling its flower or leaves.

**Figure 2a and 2b:**
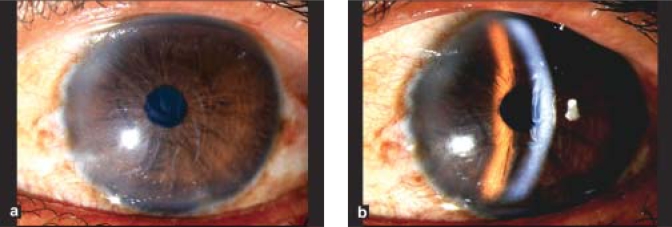
Corneal edema and severe Descemet's folds in a patient after 48 hours

There was a strong male preponderance (83%), with only five female patients. Sixteen (55%) patients were flower vendors by profession and 21 (72%) patients had presented within 24 h of the injury. Right eye was affected in 25 (86%) cases [Table 1]. All eyes showed mild conjunctival congestion and mild to moderate ciliary congestion. All eyes had mild to severe corneal edema with Descemet's folds [Fig. 2a,2b], nine (31%) had mild to moderate degree of iridocyclitis, and seven (24%) had secondary glaucoma. The range in intraocular pressure rise in these eyes as recorded by NCT was between 8.0–11.5 mm of Hg as compared to the fellow normal eye. In 27 (93%) eyes, the mean period of complete resolution was 6.69 ± 2.70 days (range: 3–14 days) and the treatment was discontinued one week after the recovery. One case with large abrasion required bandage contact lens and complete resolution occurred after four weeks. The post-treatment BCVA returned to 20/20 in 26 (90%) cases with normal appearance of the cornea in all cases. In 17 (74%) out of 23 eyes, specular microscopy revealed a mean low cell count of 18.63 ± 9.91% after a period of three to six months in comparison to the normal fellow eye (*P* < 0.001) [Fig. 3a,3b].

**Figure 3a:**
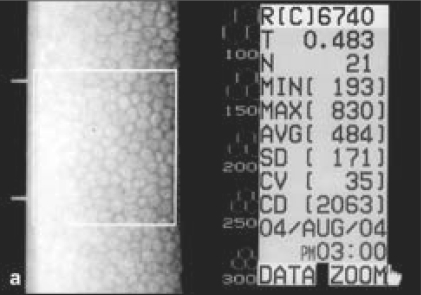
Right eye of one patient: specular microscopy shows normal endothelial cell count and morphology

**Figure 3b:**
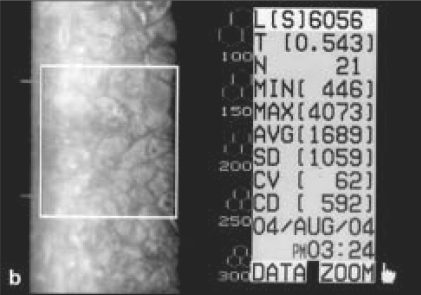
Left eye of the same patient six month after the injury: specular microscopy shows polymegathism and pleomorphism with low endothelial cell count and increased corneal thickness

**Table 1:** Patients' profile (n = 29) with presenting clinical features

Patients' profile and clinical features	No. of patients (%)
Age (year)	24–71 (Mean: 41.3 ± 9.3)
Gender (Male: Female)	24:5
Right eye: Left eye	25:4
Occupation:	
Flower vendor:	16 (55.2%)
Brahmin Pundit:	7 (24.1%)
Household person:	6 (20.7%)
Time of presentation:	
Within 24 h	21 (72.4%)
24 – 48 h	7 (24.1%)
After 48 h	1 (03.5%)
Chief complaints:	
Sudden dimness of vision	29 (100%)
Photophobia	29 (100%)
Visual acuity on presentation:	
<20/200	8 (27.6%)
20/200 to <20/60	17 (58.6%)
20/60 to 20/40	4 (13.8%)
Corneal edema with Descemet's folds	29 (100%)
Epithelial defect	3 (10.3%)
Iridocyclitis	9 (31.0%)
Secondary glaucoma	7 (24.1%)

## Discussion

The latex of *Calotropis procera* contains several alkaloids (such as *Calotropin, Catotoxin, Calcilin, Gigantin)* which are caustic and considered poisonous in nature.[2,8] Previous reports showed that accidental contact of *Calotropis* latex into the eye caused violent kerato-conjunctivitis with associated corneal edema and gross dimness of vision but without any pain.[5–7,9] But they did not report any uveitis or secondary glaucoma in any case. However, confocal microscopy of a recently reported case showed permanent endothelial cell damage which was evident after three weeks.[7]

In this series, all patients presented with sudden dimness of vision with photophobia due to corneal edema with Descemet's folds. Again as with other reports, pain was surprisingly absent in all the cases. This kind of painless course is probably due to the anesthetic properties of *Calotropis* latex.[3] Seventeen out of 24 eyes showed significant low endothelial cell count after three months follow-up compared to the fellow normal eye. These findings suggest that unlike other chemical burns, *Calotropis* latex is paradoxically relatively non-toxic to the corneal epithelium, but highly toxic to the corneal endothelium. That is why there was corneal edema with varying degree of Descemet's folds. The management in most cases is as simple as treating a mild chemical burn.

In conclusion, the latex of *Calotropis procera* causes immediate severe corneal damage with painless sudden dimness of vision. It may also cause reduction in endothelial cell count over a period of time. Every year during the months of February and April, a few people suffer from this kind of chemical injury during the festive season. Simple health education like washing of hands, avoiding contact or rubbing of eyes while plucking of *Calotropis* flower is important to prevent this kind of injury.
